# Prevalence and risk factors of domestic violence in women attending the National Guard Primary Health Care Centers in the Western Region, Saudi Arabia, 2018

**DOI:** 10.1186/s12889-020-8156-4

**Published:** 2020-02-17

**Authors:** R. Wali, A. Khalil, R. Alattas, R. Foudah, I. Meftah, S. Sarhan

**Affiliations:** 1 Ministry of National Guard-Health Affairs, Jeddah, Saudi Arabia; 2 King Abdullah International Medical Research Center, Jeddah, Saudi Arabia; 3 King Saud Bin Abdulaziz University For Health Sciences, Jeddah, Saudi Arabia

**Keywords:** Domestic violence, Women abuse, Primary health care, Prevalence, Risk factors, Saudi Arabia

## Abstract

**Background:**

Domestic violence (DV) is considered a public health issue in Saudi Arabia as well as a violation of a fundamental human right. DV causes many acute and chronic physical and mental health consequences. Cultural taboos and lack of awareness regarding the appropriate support services can increase the number of cases annually.

The objective of the study was to assess the prevalence and risk factors of DV in women attending the National Guard Primary Health Care Clinics in the Western Region of Saudi Arabia.

**Methods:**

A cross-sectional study was conducted with patients attending five Primary Health Care Centers in Jeddah from August 2017 to February 2018. A convenient sampling method was used. In total, 1845 participants were invited to complete a self-report validated Arabic version of the Norvold Domestic Abuse Questionnaire (NORAQ) to determine the prevalence and risk factors of DV. All women between 18 and 65 years who met the inclusion criteria were included. The data were analyzed using SPSS (Statistical Package Social Sciences) version 24.0.

**Results:**

The lifetime prevalence of DV in the study sample was 33.24%, with psychological abuse the most prevalent (48.47%), followed by physical abuse (34.77%) and sexual abuse (16.75%). A small proportion (4.1%) suffered from all three types of abuse. Risk factors for being a victim of abuse include being single or divorced, having a postgraduate level of education, employed, and being financially independent of the husband.

**Conclusion:**

DV is prevalent in Saudi Arabia. Modernization has shifted the risk factors, identifying the risk factors and victim characteristics would support the development and implementation of preventive and screening programs to facilitate the early identification of cases as well as the initiation of empowerment programs for Saudi women.

## Summary Boxes

### What is already known on this subject?

Domestic violence is prevalent in Saudi Arabia. Cultural Taboos play an essential role in the occurrence of abuse. All types of Domestic Violence are prevalent in Saudi Arabia, and women are reluctant to disclose being abused due to several factors, one of which is saving the family from destruction and feeling ashamed. For the last 2 years, with the new Saudi Vision (2030), different taboos have been changed, and women are more empowered and able to decide on her decisions and well-being.

### What does this study add?

This study is the first study that demonstrated that risk factors for domestic violence could change with the change in population demographics as well as with modernization. It is essential to know about the current risk factors of DV to address proper educational, screening, and preventive programs. This study also demonstrates that, with the change of the DV risk factors, the prevalence of domestic violence remains the same, which lets us think of developing interventions that aim to decrease the prevalence like public educational programs and screening programs for women presenting to the primary care centers.

## Background

Domestic Violence (DV) is defined as violent behavior perpetrated by a member of a family against another to humiliate, dominate, and govern the person’s behavior [1–3]. Violent behavior has many forms, including physical (direct or indirect), sexual, psychological, or emotional [2]. Although some societies do not recognize DV as a social issue, it is a global phenomenon as it breaches fundamental human rights [4].

DV against women has become a global problem and is prevalent in both Western and Arab countries. As reported by the World Health Organization (WHO), 29.8% of women in America and 25.4% in the European regions have been victims of physical and/or sexual abuse by a partner or sexual abuse by a non-partner [5]. In addition, 1 in 3 women in Palestine, Egypt, Tunisia, and Israel was domestically abused in 2003–2005 [6, 7]. Globally, approximately one- third of women experienced DV. Societies that do not recognize DV as a problem may overlook the physical, emotional, and social consequences of the victims, their families, and society as a whole, with suicide attempts being the most recent consequence [4, 8].

Saudi Arabia is predominantly a men-dominated culture, and men may exhibit controlling behavior towards women. There are many cultural taboos within Saudi families, and a woman is responsible for taking care of her family and preventing family destruction, even if she is unhappy in the relationship. These taboos may underpin why women avoid asking for assistance or report abuse to her family or doctors. Recently, the role of women in the community has been a topic of discussion, and DV against Saudi women has been highlighted. In 2019, several cases have been reported of Saudi women fleeing the country due to being physically or emotionally abused [9]. At present, DV is recognized as a public health and human right concern in Saudi society.

Several studies have been conducted in Jeddah, Al Madinah, Al-Ahsa, and Riyadh in Saudi Arabia, to estimate the prevalence of DV [10–13] In AL Madinah in 2012, the prevalence of ever-abused women was 34% [10]. In the Eastern Region of Saudi Arabia, Al-Ahsa, the lifetime prevalence of DV against women ranged from 39.3 to 57.7% in 2010 [11]. In Jeddah, the prevalence was estimated to be 34% [12]. In Riyadh, Saudi married women had a lifetime prevalence of 43.0% for any type of violence [13].

The scope of this dilemma has not been investigated in the National Guard population in the Western Region of Saudi Arabia. The study aimed to determine the prevalence and risk factors of DV in women and attendees of the National Guard Primary Health Care clinics in the Western Region of Saudi Arabia. This study could serve as the foundation for further studies and culture-specific screening programs in the future.

## Methods

This research was an across-sectional study conducted from 2017 to 2018, in the National Guard Primary Healthcare Centers in the Western Region. The centers include Iskan, Bahra, the Specialized Polyclinic in Jeddah, Alsharea clinic in Makkah, and Iskan clinic in Taif. All are satellite clinics of King Abdulaziz Medical City (KAMC).

The sample size was calculated at a 95% confidence interval (CI) level with a 50% response distribution and a margin of error of ±5%. The required sample size was determined to be 1845 using Raosoft software (http://www.raosoft.com/samplesize.html).

A non-probability convenient sampling technique was used to select study participants. Women patients and attendees, age 18 to 65 years, were invited to complete a self-administered questionnaire. Trained data collectors interviewed participants who could not read or write. Critically ill women were excluded from enrollment.

The validated Arabic version of the Norvold Domestic Abuse Questionnaire (NORAQ) was used. It is a questionnaire that can be used to study the prevalence of DV in addition to the risk factor. It also highlights different types of abuse, severity, and perception of victims about their health. The Arabic version was also validated and made it more useful in collecting data about sensitive issues. The Arabic NORAQ total content validity index is 0.90, while the alpha reliability coefficients were 0.75 for the total scale and ranged from 0.75 to 0.77 for the subscales.

The questionnaire measures three types of abuse: psychological, physical, and sexual [14].

The questionnaire had five sections. The first section included questions regarding the participant’s general health status as well as her socio-demographic characteristics, including age, level of education, religion, marital status, and age when married, and if married, whether she was divorced, or widowed. Also included were questions related to the participant’s employment status, household income, and financial dependence on her husband. The participant’s health status was explored in terms of the frequency of hospital visits and admissions in the past year, as well as any somatic symptoms or symptoms of depression or insomnia.

The second, third, and fourth sections consisted of questions regarding emotional, physical, and sexual abuse. The participants were primarily asked about a history of abuse. If DV occurred, she was asked to continue to answer more questions that are detailed. The questions focused on the age she was initially abused, abuse within the last year, the extent of the impact of the abuse on the patient’s life, the identity of the abuser, and if the abuser was abusing substances. Besides, disclosure and assistance seeking behavior were explored. The last part of the questionnaire explored the patient’s attempts to receive assistance, awareness of the institutes offering support services, and being fearful and at risk of being abused.

Ethical approval for the study was obtained from the Institutional Review Board (IRB) of King Abdullah International Medical Research Center (KAIMRC). Ethical principles were maintained throughout the research process. All participants signed informed consent, and they were informed about their right to withdraw from the study. Confidentiality and anonymity were assured. The data were stored in workplace computers with access to only the researchers. The investigators were available to answer any questions about the questionnaire during data collection.

The data were analyzed using SPSS (Statistical Package Social Sciences) version 24.0. Continuous variables are presented as mean and standard deviation and categorical variables as frequency and percentage. For inferential statistics, Pearson’s Chi-square was used to test the relation between DV and the qualitative independent variables. The T-Test was used to test the relationship between DV and the quantitative independent variables. A *p*-value < 0.05 was considered significant.

## Results

### Demographic characteristics

The sample size realized as 1845 participants aged between 18 and 65 years. All were Saudi nationals, belonged to the Muslim religion and dependents of National Guard employees. The mean age of the sample was 32.24 ± 10.92 years. Married women constituted 71.96% (*n* = 1304) of the sample, with the mean duration of marriage 1.80 ± 1.15 years, and the mean age when married 16.87 ± 9.44 years. The proportion of single women were 22% (*n* = 406) with smaller proportions of divorced women 2.5%, (*n* = 46) and being widowed 3.09%, (*n* = 56) (Table 1).

**Table 1:** Demographic characteristics of the sample

a		
Variable	Mean	SD
Age (*n* = 1810)	32.246	10.927
Age at marrige (*n* = 1703)	16.86	9.442
Duration of marriage (*n* = 1717)	1.801	1.152
Family members (*n* = 1801)	6.13	3.178
Number of rooms (*n* = 1700)	5.111	1.9071
b		
Variable	Frequency	%
Residence (*n* = 1845)		
● UK (unknown)	20	1.11
● City	1503	83.13
● Village	285	15.76
Financially dependent (*n* = 1383)		
● Yes	1074	77.65
● No	309	22.34
Marital status (*n* = 1812)		
● Single	406	22.41
● Married	1304	71.96
● Divorce	46	2.54
● Widow	56	3.09
Education level (*n* = 1808)		
● Illiterate	182	10.06
● Undergraduate	826	45.66
● Graduate	770	42.56
● Postgraduate	31	1.71
Residence (*n* = 1806)		
● Owner	1081	59.86
● Rent	725	40.14
Employment status (*n* = 1813)		
● Student	5	0.28
● Employed	258	14.23
● Housewife	1546	85.27
● Retired	4	0.22
Income		
● less than 5000	639	35.42
● More than 5000	1165	64.58

In terms of accommodation, the majority of the study population 83.13% (*n* = 1503), lived in the city with 15.76% (*n* = 285) living in the village. More than half 59.86% (*n* = 1081) owned their own home and 40.14% (*n* = 725) rented accommodation. The mean number of rooms per dwelling was 5.11 ± 1.90, and the mean number of family members 6.13 ± 3.178 (Table 1).

Regarding financial dependence on the caregiver (*n* = 1383), more than half 77.65% (*n* = 1047) were financially dependent and a small proportion 22.34% (*n* = 309) were financially independent. Where the majority (64.58%, *n* = 1165) receives monthly income more than 5000 SR, with a small proportion 35.42%, (*n* = 639) receives less than 5000 SR. In terms of the educational level of the sample (*n* = 1808), a small proportion was illiterate 10.06% (*n* = 182), undergraduate 45.66% (*n* = 826), graduate 42.56% (*n* = 770) and postgraduate 1.71% (*n* = 31). The majority (*n* = 1813) were housewives 85.27% (*n* = 1546), with a small proportion employed 14.23% (*n* = 258) and 0.22% (*n* = 4) retired.

### Prevalence of domestic violence

**Fig. 1:**
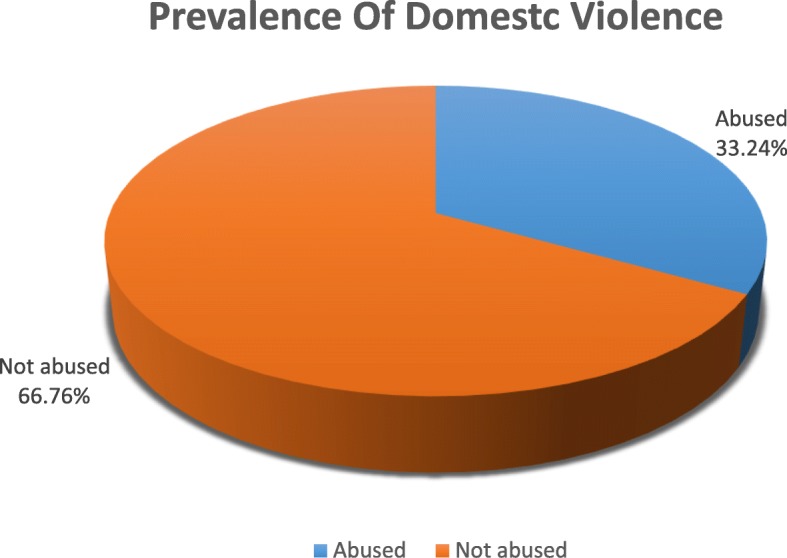
Prevalence of Domestic Violence

**Fig. 2:**
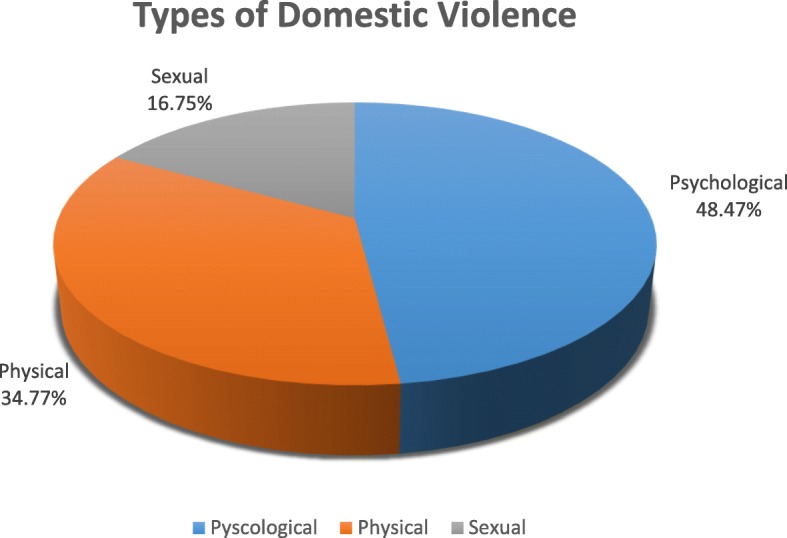
Domestic Violence by Type

The lifetime prevalence of DV in the sample (*n* = 1802) was 33.24% (*n* = 599) (Fig. 1). Psychological abuse was the most prevalent 48.47% (*n* = 460) followed by physical abuse 34.77% (*n* = 330) and a smaller proportion 16.75% (*n* = 159) reported sexual abuse. A small proportion 4.1% (*n* = 75) reported suffering all three types of abuse. (Tables 2 and 3), (Fig. 2).

**Table 2:** Prevalence of Abuse in Sample

Prevalence of abuse	Abused		Not abused	
	n	%	n	%
Ever abused (*n* = 1802)	599	33.24	1203	66.76
All type of abuse (*n* = 1813)	75	4.1	1738	95.9

**Table 3:** Domestic Violence by Type

Type of abuse	Abused	
	n	%
Psychological	460	48.47
Physical	330	34.77
Sexual	159	16.75

### The relationship between demographic characteristics and abuse status in the sample

Table 4 displays a significant relationship between abuse status and level of education (*p* < 0.005). The abused status is most prevalent in participants with a postgraduate education 38.7% (*n* = 12) followed by a graduate-level education 38.2% (*n* = 292), undergraduate education 30.7% (*n* = 252) and 23.5% (*n* = 42) were illiterate. Being employed was also statistically significant (*p* < 0.005). The prevalence of abuse was highest among employed women 45.9% (*n* = 118) compared to housewives or retired women. The place of residence, type of home, and family income were not statistically significant.

**Table 4:** The Relationship between Demographic Characteristics and Abuse Status

Variable	No	Abuse No (%)	No Abuse No (%)	^a^Chi- square	*p*-value
Employment status					
● Employed	257	118 (45.9)	139 (54.1)	22.422	.000
● Housewife	1536	477 (31.1)	1059 (68.9)		
● Retired	4	2((50)	2 (50)		
Educational level					
● Illiterate	179	42 (23.5)	137 (76.5)		
● Undergraduate	822	252 (30.7)	570 (69.3)		
● Graduate	765	292 (38.2)	473 (61.8)	19.597	.001
● Postgraduate	31	12 (38.7)	19 (61.3)		
Residence					
● Unknown	19	8 (42.1)	11 (57.9)		
● City	1495	490 (32.8)	1005 (67.2)	1.773	.635
● Village	283	100 (35.3)	183 (64.7)		
Home type					
● Owned	1074	367 (34.2)	707 (65.8)	1.002	.317
● Rent	721	230 (31.9)	491 (68.1)		
Family income					
● < SAR 5000	633	213 (33.6)	420 (66.4)	.061	.805
● > SAR 5000	1161	384 (33.1)	777 (66.9)		
Social status					
● Single	404	158 (39.1)	246 (60.9)		
● Married	1295	394 (30.4)	901 (69.6)	29.330	.000
● Divorce	46	29 (63)	17 (37)		
● Widow	56	18 (32.1)	38 (67.9)		
Marriage duration					
● < 5 years	362	120 (33.1)	242 (66.9)		
● 5–10 years	349	108 (30.9)	241 (69.4)	8.11	.044
● ≥ 10 years	671	205 (30.6)	466 (69.4)		
Financial dependence on Alhusband					
● Yes	1065	298 (28)	767 (72)	21.191	.000
● No	309	129 (41.7)	180 (58.3)		

^a^Chi-Square Test

The social status was significantly associated with abuse, and most prevalent in the divorced group 63%, (*n* = 29) followed by being single 39.1% (*n* = 158), widowed 32.1% (*n* = 18), and lowest in the married group 30.4% (*n* = 394). Being financially independent of the husband 41.7% (*n* = 129), was also statistically significant. The duration of marriage had no significant association with the abuse status.

### Psychological abused participant characteristics and source of abuse

This study estimates the prevalence of psychological abuse at 48.47% (*n* = 460). The mean age when first abused was 13.07 ± 11.27 years and the mean age of informing another person about being abused 14.86 ± 11.63 years. The mean score on a 10 point scale (0 = no suffering, 10 = suffering terribly) for current suffering in the abused group was 5.34 ± 2.98. About a third of the sample 33.92% (*n* = 153) did not attempt to find assistance as they did not consider themselves to be suffering, 39.46% (*n* = 178) did not attempt to find assistance though they were suffering and only a small proportion 26.6% (*n* = 128) attempted to obtain assistance. (Table 5).

**Table 5:** Psychologically abused participant characteristics and source of abuse

Variable	Mean	SD
Age at first psychological abuse (*n* = 384)	13.07	11.27
Mean score of abuse consequence (*n* = 425)	5.34	2.986
Age of disclosure (*n* = 210)	14.85	11.630
Variable	Frequency	%
Psychological abuse in the last year (*n* = 450)		
● No	303	67.33
● Yes	147	32.66
Seeking assistance (*n* = 451)		
● No, did not suffer	153	33.92
● No, although suffering	178	39.46
● Yes	120	26.60
Source of abuse (*n* = 602)		
● Ex-husband	31	5.14
● Current husband	98	16.27
● Stepfather	5	0.83
● Stepmother	19	3.15
● Mother	52	8.63
● Father	93	15.44
● Brother	82	13.62
● Sister	34	5.64
● Children	24	3.98
● Friends	38	6.31
● Known person	43	7.14
● Unknown person	26	4.31
● Others	57	9.46
What do abusers use (*n* = 371)		
● Smoking	140	37.73
● Drugs	17	4.58
● Alcohol	8	2.156
● Nothing	206	55.52
Disclosure		
● No	251	55.29
● Yes, partially	140	30.84
● Yes, all	63	13.88
Disclosure to a medical practitioner (*n* = 230)		
● No	195	84.78
● Yes, she knows	5	2.17
● Yes, when she asked	17	7.39
● Yes, immediately	13	5.65

Of the psychologically abused group, 32.66% (*n* = 147) were psychologically abused in the last year and the abuser was the current husband 16.27% (*n* = 98), father 15.44% (*n* = 93), brother 13.62% (*n* = 82), mother 8.63% (*n* = 52), a person known by the family 7.14% (*n* = 43), and friends 6.31% (*n* = 38). According to participants, 37.73% (*n* = 140) of the abusers were smokers, 4.58% (*n* = 17) were drug users, and 2.156% (*n* = 8) were alcoholics. (Table 5).

In terms of disclosure, more than half 55.29% (*n* = 251) did not disclose the abuse at all, 30.84% (*n* = 140) partially disclosed, and only 13.88% (*n* = 63) disclosed the abuse to another person. Regarding disclosing to a medical practitioner, 84.78% (*n* = 195) did not disclose at all, 2.17% (*n* = 5) thought her doctor knew, 7.39% (*n* = 17) disclosed only when the doctor inquired, and 5.65% (*n* = 13) disclosed immediately. (Table 5).

### Physical abused patient characteristics and source of abuse

The prevalence of physical abuse in this study was 330 (34.77%). The mean age when first abused was 12.79 ± 10.09 years, while the mean age when informing a person about being abused was 13.63 ± 10.57 years. The mean score on a10 point scale (0 = no suffering, 10 = suffering terribly) for the current situation of the abused participants was 4.79 ± 2.814. (Table 6).

**Table 6:** Physically abused participant characteristics and source of abuse

Variable	Mean	SD
Age at first physical abuse	12.79	10.094
Mean score of physical abuse consequence	4.79	2.814
Age of disclosure	13.62	10.572
Variable	Frequency	%
Physical abuse in the last year (*n* = 322)		
● Yes	96	29.81
● No	226	70.18
Seeking assistance (*n* = 323)		
● No, did not suffer	164	50.77
● No, although suffering	92	28.48
● Yes	67	20.74
Source of abuse (*n* = 228)		
● Ex-husband	22	9.6
● Current husband	55	24.12
● Stepfather	3	1.31
● Stepmother	5	2.19
● Mother	15	6.57
● Father	40	17.54
● Brother	41	17.98
● Sister	2	0.87
● Children	3	1.31
● Friends	14	6.14
● Known person	11	4.82
● Unknown person	3	1.31
● Others	14	1.75
Substance abuse of abusers (*n* = 260)		
● Smoking	112	43.07
● Drugs	13	5
● Alcohol	9	3.46
● Nothing	126	48.46
Disclosure (*n* = 321)		
● No	175	54.51
● Yes, partially	98	30.52
● Yes, all	48	14.95
Disclosure to medical practitioner (*n* = 179)		
● No	144	80.44
● Yes, she knows	3	1.67
● Yes, when she asked	20	11.17
● Yes, immediately	12	6.70

Of the physically abused group, 29.81% (*n* = 96) were abused in the last year. Half of the physically abused women 50.77% (*n* = 164) did not attempt to obtain assistance because they thought they were not suffering, 28.48% (*n* = 92) did not attempt to be assisted though they were experiencing suffering and 20.74% (*n* = 67) attempted to be assisted. (Table 6).

The source of abuse was the current husband 24.12% (*n* = 55), father and brother 17.54% (*n* = 40) and 17.98% (*n* = 41) respectively, ex-husband 9.6% (*n* = 22) and friends 6.14% (*n* = 14). Some of the abusers were smokers 43.07% (*n* = 112), drug abusers 5% (*n* = 13) and 3.46% (*n* = 9) abused alcohol.

In terms of disclosure, 54.51% (*n* = 175) did not disclose being abused, 30.52% (*n* = 98) partially disclosed and only 14.95% (*n* = 48) informed another person. Regarding informing their medical practitioner, 80.44% (*n* = 144) did not disclose, 1.67% (*n* = 3) thought their doctor knew, 11.17% (*n* = 20) disclose to the doctor when asked about abuse and 6.7% (*n* = 12) immediately disclosed to her doctor. (Table 6).

### Sexual abused participants’ characteristics and source of abuse

The prevalence of sexual abuse was 16.75% (*n* = 159). The mean age of the first experience of sexual abuse was 9.92 ± 7.63 years, and the mean age of disclosing the abuse was 11.92 ± 8.49 years. The mean score on a 10 point scale (0 = no suffering, 10 = suffering terribly) for the current level of suffering 5.307 ± 3.02. (Table 7).

**Table 7:** Sexually abused participant characteristics and source of abuse

Variable	Mean	SD
Age at first sexual abuse	9.92	7.633
Mean score of sexual abuse consequence	5.307	3.0282
Age of disclosure	11.92	8.492
Variable	Frequency	%
Sexual abuse in the last year (*n* = 136)		
● Yes	20	14.7
● No	116	85.29
Seeking assistance (n = 138)		
● No, did not suffer	67	48.55
● No, although suffering	54	39.13
● Yes	17	12.31
Source of abuse (*n* = 117)		
● Ex-husband	6	5.12
● Current husband	15	12.82
● Stepfather	1	0.85
● Stepmother	1	0.85
● Mother	0	0
● Father	5	4.27
● Brother	17	14.52
● Sister	1	0.85
● Children	1	0.85
● Friends	6	5.12
● Known Person	27	23.07
● Unknown Person	15	12.82
● Others	22	18.80
Substance abuse of abusers (*n* = 110)		
● Smoking	53	48.18
● Drugs	5	4.54
● Alcohol	6	5.45
● Nothing	46	41.81
Disclosure (*n* = 135)		
● No	88	65.18
● Yes, partially	30	22.22
● Yes, all	17	12.59
Disclosure to a medical practitioner (*n* = 56)		
● No	49	87.5
● Yes, she knows	1	1.78
● Yes, when she asked	5	8.92
● Yes, immediately	1	1.78

Of the sexually abused group, 14.7% (*n* = 20) were abused in last year. About half of sexually abused group 48.55% (*n* = 67) did not attempt to obtain assistance as, in their opinion, they were not suffering, 39.13% (*n* = 54) did not attempt to find assistance though they were suffering and only 12.31% (*n* = 17) attempted to find assistance. (Table 7).

The source of the sexual abuse was a known person 23.07% (*n* = 27), others 18.8% (*n* = 22), brother 14.52% (*n* = 17), an unknown person, and current husband, both 12.82% (*n* = 15). Some of the abusers were smokers 48.18% (*n* = 53), while 4.54% (*n* = 5) were drug abusers, and 5.45% (*n* = 6) were abusing alcohol.

Regarding disclosure 65.18% (*n* = 88) did not disclose the abuse, 22.22% (*n* = 30) partially disclosed, and only 12.59% (*n* = 17) disclosed the abuse completely. In addition, 87.5% (*n* = 49) did not disclosed to the doctor, 1.78% (*n* = 1) thought her doctor knew, 8.92% (*n* = 5) disclosed to her doctor when asked about abuse, and only 1.78% (*n* = 1) fully disclosed to her doctor. (Table 7).

## Discussion

DV is a public health issue, both nationally and internationally, and causes many adverse physical and mental health consequences. The prevalence of DV varies between countries depending on cultural taboos and the definition of violence. A systematic review conducted in Turkey reported that DV is prevalent in the country [15]. The estimated prevalence of DV in Arab countries, reported in a systematic review, was 73.3%, with 35.6% physical, 49.8% psychological, and 22.0% sexual abuse [16].

The current study estimated the prevalence of DV in the Western Region of Saudi Arabia, including Makkah, Jeddah, and Al-Taif 33.24%. The prevalence is similar to a study conducted in 2017, including 758 Saudi Arabian women from 13 governorates in Saudi Arabia, with a reported prevalence of 32% [17]. The reality, based on the current and previous studies, is that at least one-third of Saudi women is a victim of abuse. The prevalence of DV in the National Guard population in the western region of Saudi Arabia is similar to the prevalence of DV conducted in the university hospital in the same area [17] meaning that the prevalence of DV could be consistent across different sectors regardless of the changing risk factors.

The current study estimated the prevalence of psychological (48.47%), physical (34.77%), and sexual abuse (16.75%). Compared with studies done in Saudi Arabia, sexual abuse had a lower prevalence, but physical and psychological abuse was more prevalent [10–12]. The study indicated that women who experience suffering due to the abuse, had negative physical or mental health consequences, adversely affecting the victim’s life [18].

Specific socio-demographic characteristics are associated with an increased or decreased risk of DV. For example, in terms of educational level, in a study done in West Bengal, the highest prevalence of DV was in the illiterate group (46.15%) [19]. A study conducted in Sevas, Turkey, also reported the highest prevalence of DV in the illiterate group [20]. In contrast, the current study concluded that the postgraduate education group had the highest prevalence of abuse, as also reported in the study conducted in Riyadh [21]. Possible explanations could be that the higher education level is associated with financial independence, a higher awareness of DV, and the different types of abuse, enabling women to consider particular behavior as acceptable or tolerable. Besides, the abuser could feel threatened and unsafe with educated women. Employed women also had a higher prevalence of abuse compared to housewives which are similar to another study conducted in Jeddah during the year 2002 [12], which could be due to the same reasons. Lastly, the difference could be cultural and the availability of a reporting system in both countries. No studies, according to the researcher’s knowledge, explained this hypothesis.

Another socio-demographic factor, financial dependence on a husband, reduced the risk of DV compared to financially independent women. In contrast, a study done in Jeddah reported that women, who were financially dependent on a husband, had 1.5-fold odds of being physically abused by the husband [12].

In the current study, women were abused at a young age, but they did not report the abuse to another person until several years later. It is noteworthy that more than half of the victims of psychological and physical abuse were still being abused in the last year, while sexual abuse tends to decrease as the woman matures.

All most all of the victims of all types of abuse are reluctant to seek assistance 97.2%. Possible reasons could be due to being in a conservative culture and fear of shame, normalizing some of the acts of abuse in a predominantly male culture, or because they do not know where to find assistance. The results of the study emphasize the need to create awareness, teach women how to say no for abuse, and better understand the concept of abuse and screening programs. Another important aspect is the role of social stigma, which may have rendered the participants who were interviewed, mostly the illiterate and undergraduate groups, fearful of exposing such information. A study conducted in the USA reported that abused women are reluctant to report being abused due to feeling unsafe, fear of more severe abuse if the abuser found out, or an unclear reporting pathway [22].

In both psychological and physical abuse, the abuser was the husband or ex-husband if the victim was married and the father or brother if she was not married. Sexual abuse was perpetrated by the brother or a person known to the family. According to the Center for Disease Control and Prevention, risk factors for being abused are witnessing DV as a child, living in a controlling relationship, low income, emotional and financial dependency, and being abused as a child [23].

From the current study, abusive behaviors were common among substance abusers, with smoking most prevalent, followed by drugs and alcohol. The finding is similar to a study conducted in India, where abusers are commonly found to be substance abusers [24]. The findings support the development of a drug-screening program to decrease the incidence of DV.

### Study limitations

There may be some possible limitations in this study. For example, sampling bias, where the selected population may not represent the real population under study which can affect the generalizability of the results. The sensitivity of the questions in the questionnaire is another limitation because it addresses sensitive issues like sexual abuse. Being afraid of stigma or labeling was another issue, although it was addressed in the consent form, some participants may still be hesitant to answer. Not only that, some questions were not answered thoroughly, which change the total number of responders from one question to another.

To overcome this limitation in the future, this research can be conducted on another population to help in generalizing the results. Second, using an online questionnaire may help to have more responders and complete answers.

### Implications for practice and research

This study showed that Domestic violence is prevalent in Saudi Arabia, like other parts of the world. Besides, it showed that risk factors for being abused are changing. With this new information, stakeholders need to address those factors by health education and screening programs.

This study also showed that women would not volunteer the information about being abused until she was asked by a health professional, which emphasizes the need for screening programs.

It is the first Saudi study that shows a shift in the risk factors for domestic violence; the same study should be conducted on different populations to confirm the findings.

## Conclusion

DV, in all its forms, is prevalent in the women attending the National Guard Primary Health Care Clinics, as well as globally. With the rapid social openness in the form of allowing women to drive cars, to be independent after the age of 18 and full custody of her children, and much more, there is a shift in the risk factors for being a victim of DV. Culture-specific preventive and screening programs can contribute to reducing contributing factors for perpetrating abuse, supporting a reduction in the prevalence of abuse in the Saudi community.

## Data Availability

All data generated or analysed during this study are included in this published article.

## Abbreviations

DV: Domestic violence
KAMC: King Abdulaziz Medical City
NORAQ: Norvold Domestic Abuse Questionnaire
WHO: World Health Organization
